# Intra-operative blood transfusions raise the risk of postoperative delirium and impede functional recovery in elderly hip fracture patients: a propensity score-matched study

**DOI:** 10.1186/s10195-025-00825-2

**Published:** 2025-02-28

**Authors:** Yanan Li, Tao Wang, Jiajie Zhang, Zhiqian Wang, Junfei Guo, Qi Zhang

**Affiliations:** 1 Department of Anesthesiology, The Third Hospital of Hebei Medical University, Hebei, China; 2 Department of Lower Limb Trauma, Beijing Jishuitan Hospital, Guizhou Hospital, Guizhou, China; 3 Department of Anesthesiology, Hebei Children’s Hospital, No.133 Jianhua South Street, Shijiazhuang City, Hebei, 050031 China; 4 Department of Orthopaedic Surgery, Third Hospital of Hebei Medical University, Hebei, China; 5 Orthopaedic Research Institute of Hebei Province, Hebei, China; 6 NHC Key Laboratory of Intelligent Orthopaedic Equipment (The Third Hospital of Hebei Medical University), Hebei, China; 7 Department of Joint Surgery, Honghui Hospital, Xi’an Jiaotong University, No.555 Youyi East Road, Xi’an City, 710001 China; 8 Graduate School, Hebei Medical University, Shijiazhuang, China

**Keywords:** Blood transfusion, Elderly, Intertrochanteric fractures, Postoperative delirium, Postoperative outcome, Propensity score matching, Retrospective study

## Abstract

**Background:**

This retrospective analysis was performed to investigate the potential influence of intra-operative blood transfusion (IBT) in patients aged 65 years or older with intertrochanteric fractures (IF) who underwent intramedullary fixation.

**Methods:**

The outcomes of interest included the incidence of postoperative delirium (POD), pain score at discharge, length of hospital stay (LOS), functional outcomes, and mortality. The study included all surgically treated patients with IF between Jan. 2018 and Dec. 2021. Data on patient demographics, injury-related factors, surgical procedures, intraoperative details, in-hospital information, and postoperative outcomes were collected. In order to mitigate potential confounding and selection bias, the researchers employed the propensity score matching (PSM) technique using a 1:1 ratio via the caliper matching method. Following PSM, the association between IBT and outcome analyses was assessed using McNemar's Chi-square tests. Additionally, the Spearman correlations between IBT, POD and postoperative functional outcomes were computed.

**Results:**

Out of the initial 2159 consecutive patients screened, a final sample of 1681 individuals was included, consisting of 1278 in the non-IBT group and 403 in the IBT group. After PSM, each group comprised 298 participants. The disparities in POD rate and functional outcomes became significant after employing propensity score-based matching (*P* < 0.001 and 0.029, respectively), despite their lack of significance prior to matching. There were no notable distinctions observed in other operation-related data, LOS, and crude mortality rates at 30-day, 90-day, and 12-month intervals before and after PSM. Furthermore, incidence of POD (*P* = 0.006) and functional outcomes (*P* = 0.013) were significantly associated with IBT.

**Conclusion:**

In conclusion, IBT significantly increases the incidence of POD and hinders postoperative functional recovery in elderly patients with hip fracture.

## Introduction

Postoperative delirium (POD) is a prevalent central nervous system complication observed in elderly patients following anesthesia and surgery [1]. It is characterized by the onset of acute delirium and subsequent long-term cognitive impairment, manifesting as mental confusion, anxiety, personality alterations, and memory deficits [2]. A multi-center retrospective study shows that individuals aged 65 and older experience POD 4–10 times more frequently than younger patients [3, 4]. Additionally, those aged 75 and older have a threefold higher incidence of POD compared to those aged 65–75 [5]. POD not only poses an elevated risk of enduring cognitive impairment and dementia among elderly patients, but also hampers their postoperative recovery, thereby imposing substantial economic and psychological burdens on both the patients and their families [6]. With the increasing elderly population and improved surgical and anesthesia methods, more elderly patients are undergoing surgeries with general anesthesia. As a result, POD and its complications have become significant medical and social issues, making the study of its pathogenesis and prevention in older adults a key research focus [7].

Despite the current lack of complete understanding regarding the etiology of POD, a substantial body of research has consistently demonstrated a strong association between increasing age, nursing home residency, pre-existing cognitive impairment, and higher American Society of Anesthesiologists (ASA) score with the occurrence of POD [8]. The prevalence of hip fractures, particularly intertrochanteric fractures, has been escalating due to the aging population and rising incidence rates, surpassing one million cases annually, particularly in developing nations [9, 10]. Consequently, hip fractures have emerged as the predominant cause of admission in orthopedic wards catering to elderly patients. As a result of numerous postoperative complications and challenges in attaining favorable outcomes, intertrochanteric fractures of the femur have emerged as a prominent public health concern among the elderly, imposing significant social and economic burdens on society [11, 12].

A recent study found that elderly patients with hip fractures require more frequent and larger intraoperative blood transfusions than other surgical groups [13]. This is due to pre-existing anemia, either present before the fracture or caused by blood loss from the fracture, and significant blood loss during procedures like reduction and fixation. Elderly individuals typically have reduced vascular elasticity and face difficulties with hemostasis [14]. They often suffer from osteoporosis and various cardiovascular and cerebrovascular conditions. Surgical treatment of fractures requires stronger internal fixation devices, like screws or steel plates, which increases surgery time and blood loss. Therefore, elderly patients with hip fractures often experience POD after surgery [11, 15]. Prompt administration of blood transfusions can enhance oxygenation capacity and sustain circulatory stability in elderly patients undergoing surgery. Nevertheless, excessive transfusion of stored blood may lead to microcirculatory dysfunction and a higher likelihood of postoperative complications. Additionally, the infusion of allogeneic blood can impede the immune regulatory function of the body, elevate the risk of myocardial ischemia, and prolong hospitalization duration [16]. Thus, we speculate that there may be a close relationship between intraoperative blood transfusion (IBT) and POD in elderly patients. Taken together, the objective of this study is to assess the impact of IBT on postoperative outcomes, including incidence of POD, length of hospital stay (LOS), functional outcomes, and mortality, in older individuals with hip fractures. The findings of this research will offer valuable insights for selecting appropriate timing for perioperative blood transfusion in elderly patients.

## Materials and methods

This retrospective study received approval from the Ethics Committee of our hospital and was registered on the Clinical Trial Registry Center. The study was conducted in compliance with the principles outlined in the Declaration of Helsinki, and consent was waived as this is an observational study without an intervention. All collected patient data were anonymously recorded to protect patient confidentiality.

### Study design, setting, and population

A retrospective analysis of IF patients was conducted at the National trauma emergency center between Jan. 2018 and Dec. 2021. The inclusion criteria for this study consisted of patients who were 65 years or older, had an ASA grade I ~ III, experienced an admission delay of less than 48 h from the initial injury, and received a minimum of 12-month follow-up. The exclusion criteria included patients with a history of central nervous system disease (such as transient ischemic attack, stroke, cerebral hemorrhage, syncope, spinal cord injury, or upright hypotension), electrolyte disturbance (including hypernatremia, hypokalemia, and hyperchloremia), a history of neurological or psychiatric disorder, currently using sedatives or antidepressants, experiencing infection or chronic inflammation, recently taking anti-inflammatory drugs, unwilling to participate in experimental procedures, suffering from speech impairment, illiteracy, hearing impairment, visual disorder, alcohol or drug addiction, or having an allergic reaction to anesthetic drugs. The patients were categorized into either the IBT or non-IBT groups based on whether they received IBT during the surgical procedure. All IBT types are packed RBCs. Patients who received autologous blood transfusion were not included in this study.

### Anesthetic management and surgical procedure

Upon the patient's entrance into the operating room, continuous monitoring of non-invasive blood pressure, pulse oxygen saturation, and electrocardiogram was conducted. Additionally, patients received a continuous inhalation of oxygen at a rate of 2 L/min via a face mask. Intraspinal anesthesia was administered in accordance with the standard protocol.

Preoperative X-rays, including anteroposterior and lateral views, as well as a Siemens 128-layer dual-source spiral CT scan (Siemens Medical System, Germany), were obtained for the injured leg. Fractures were categorized as either stable (A1.1-A2.1) or unstable (A2.2-A3.3) based on the classification system established by the Orthopaedic Trauma Association [17]. The patients underwent surgical treatment with intramedullary fixation by proximal femoral nail antirotation (PFNA), in accordance with international treatment guidelines. During the surgical procedure, the internal fixation was meticulously assessed, and the wound was subsequently sutured in a layered manner. Postoperatively, the patients were advised to gradually increase weight-bearing activities, ranging from partial to full weight bearing. To ensure proper monitoring, regular outpatient reviews or telephone interviews were conducted with either the patients themselves or their family members.

### Data collection

Data were collected retrospectively from the electronic medical record of our institution. The collected data encompassed various patient demographics such as gender, age, body mass index (BMI), residence (rural or urban), and history of smoking or alcohol use. Additionally, injury-related data included fracture type and the duration from initial injury to surgery. Surgery-related data consisted of general health status determined by the ASA grade and modified Elixhauser comorbidity measures (mECM). Lastly, inhospital data encompassed the Hb level, commonly used visual analog scores (VAS), and numerical rating scores (NRS) upon admission. Operation-related data, such as the duration of anesthesia and the duration of the operation, were collected. In-hospital outcomes, including the VAS, NRS, LOS, and incidence of POD using the Method-Chinese Revision (CAM-CR) score, were also recorded. The participants' survival status and date of death were collected during the follow-up period. The beginning of the follow-up period was defined as enrollment in the cohort, and the endpoint event was defined as death from any cause or the most recent follow-up visit, whichever occurred earlier. Additionally, 30-day, 90-day, and 12-month mortality rates, as well as functional outcomes such as independent walking, use of walking aids, wheelchair, bedridden status, and death, were also documented.

### Definitions

In this current study, the mECM derived from electronic medical records was employed to evaluate the comorbidities of patients upon admission, and subsequently categorized into groups < 0, 0, 1–5, 6–13, and ≥ 14. Furthermore, the ASA grade, which encompasses four levels, is a widely utilized assessment tool by anesthesiologists and orthopedics to evaluate a patient's physical state and surgical risk [18]. The 15-item Geriatric Depression Scale (GDS) and functional independence measure (FIM) were utilized to ascertain depression symptoms and the overall capacity to carry out daily activities, respectively [19, 20]. Investigators, blinded to patient subgroups and pre-trained, conducted return visits to apply the CAM-CR scale twice daily (8:00–10:00 a.m. and 6:00–8:00 p.m.) for 1–3 days postoperatively to assess POD. The CAM-CR score assesses 11 items: acute onset, attentional disturbance, disturbed thinking, altered awareness, disorientation, memory disturbance, perceptual impairment, psychomotor arousal and retardation, fluctuation, and sleep–wake cycle changes. Each item is rated from 1 (none) to 4 (severe), resulting in a total score between 11 and 44. A score above 22 indicates delirium.

### Statistical Analysis

To assess normality of continuous variables, the Shapiro–Wilk test was employed. For normally distributed numerical variables, the Student t test was utilized to determine group mean differences, with data presented as mean ± standard deviation (SD). In cases where data were non-normally distributed, the median and interquartile range (IQR) were reported and analyzed using the Mann–Whitney U test. Categorical variables were presented as proportions and differences were examined using either the chi-square or Fisher's exact test. In order to mitigate selection bias and potential confounding factors, propensity score matching (PSM) was employed to adjust for baseline clinical characteristics at a 1:1 ratio, with a caliper matching of 0.20. Following PSM, paired t-tests and paired chi-square tests were utilized to analyze continuous and categorical variables, respectively. Additionally, Spearman correlations were computed to examine the relationship between IBT, VAS, NRS, and relevant influencing factors. All statistical analyses were conducted using IBM SPSS Statistics for Windows, version 27.0 (IBM, Armonk, NY, USA). The level of significance was determined at *P* < 0.05.

## Results

### Demographic characters of patients

**Fig. 1:**
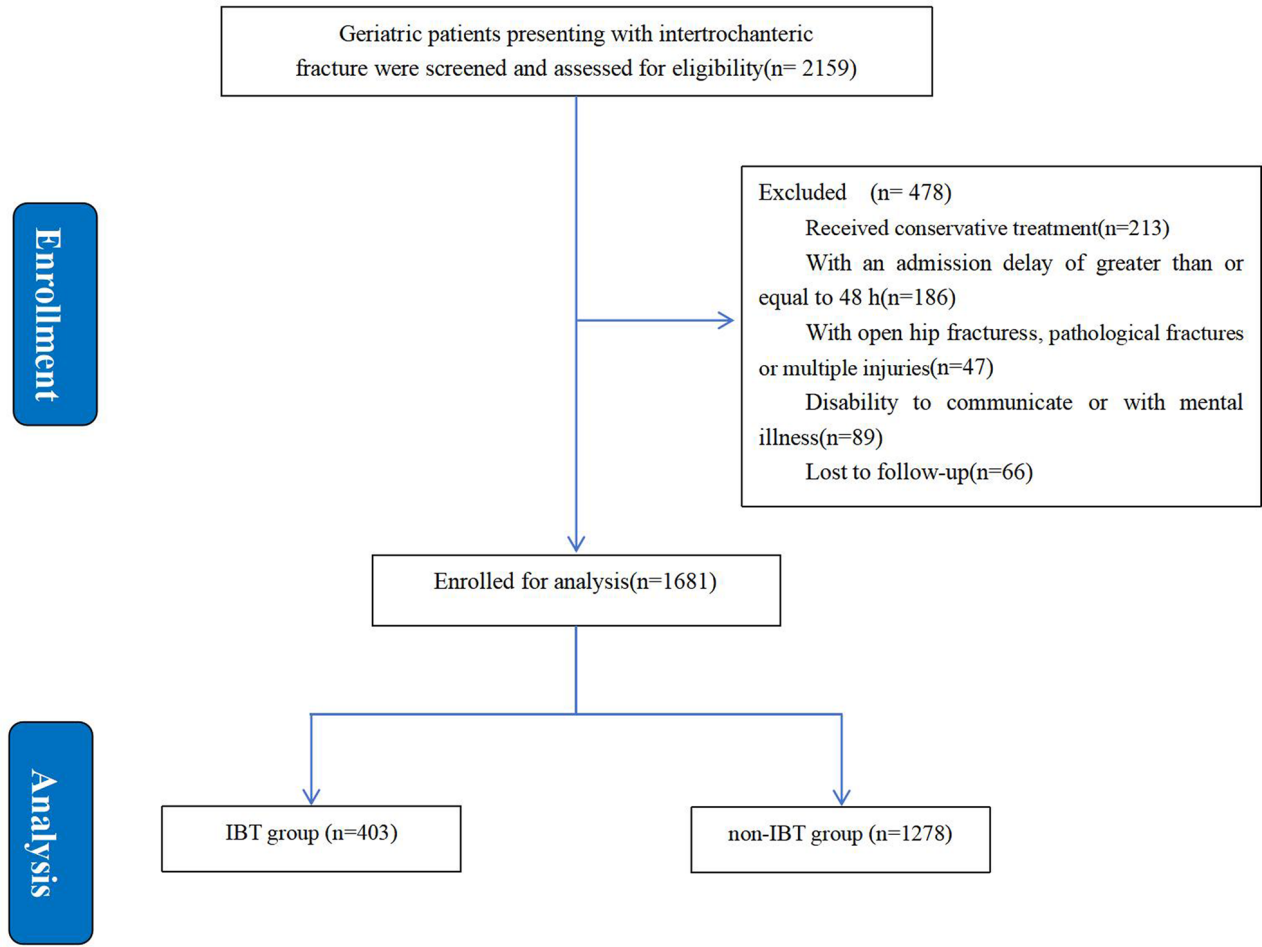
Flow diagram of the participants

Figure 1 illustrates the enrollment of study participants. From Jan. 2018 and Dec. 2021, a total of 2159 consecutive patients presenting with IF were retrospectively reviewed and assessed for eligibility. A total of 478 patients were eliminated by the exclusion criteria. Among these patients, 213 received conservative treatment; 186 had an admission delay of greater than or equal to 48 h; 47 had open hip fractures, pathological fractures, and multiple injuries; 89 had inability to communicate or with mental illness; and 66 were lost to followup. Finally, 1681 patients (including 1278 in the non-IBT group and 403 in the IBT group) who met the inclusion and exclusion criteria were enrolled.

Baseline characteristics of the patient characteristics are shown in Table 1**,** there were significant differences between the IBT group and the non-IBT group regarding gender, BMI, residence, smoking history, fracture type, time from injury to surgery, preoperative Hb, VAS, NRS, GDS and FIM at admission. There were 298 matched pairs after PSM, and the two groups had similar baseline demographic and disease characteristics (*P* > 0.05).

**Table 1:** Patient characteristics at baseline comparisons before and after propensity score matching

Variables	Pre-matching			Post-matching		
	IBT group (n = 403)	non-IBT group (n = 1278)	*P* value	IBT group (n = 298)	non-IBT group (n = 298)	*P* value
*Demographics*						
Gender [(n) %]			0.472			0.667
Male	145 (35.98%)	408 (31.92%)		107 (35.91%)	102 (34.22%)	
Female	258 (64.02%)	870 (68.08%)		191 (64.09%)	196 (65.88%)	
Age (yr)	79.86 ± 7.28	78.92 ± 7.33	0.619	79.26 ± 7.28	79.38 ± 7.28	0.703
BMI (kg/m^2^)	25.37 ± 2.65	25.84 ± 2.88	0.668	25.66 ± 2.69	25.63 ± 2.65	0.849
Residence (%)			0.027			0.589
Rural	142 (35.24%)	524 (41.00%)		109 (36.58%)	110 (36.91%)	
Urban	261 (64.76%)	754 (59.00%)		189 (63.42%)	188 (61.71%)	
Smoking history [Yes (n) %]	91 (22.58%)	226 (17.68%)	0.256	59 (19.80%)	55 (18.46%)	0.519
Drinking history [Yes (n) %]	103 (25.56%)	294 (23%)	0.189	239 (81.20%)	243 (81.54%)	0.726
*Injury-related data*						
Fracture type [(n) %]			0.013			0.476
Stable (A1.1-A2.1)	187 (46.4%)	712 (55.71%)		150 (50.33%)	154 (51.68%)	
Unstable (A2.2-A3.3)	216 (53.6%)	566 (44.29%)		148 (49.66%)	143 (48.32%)	
Time from injury to surgery (day)	6.01 ± 2.43	6.2 ± 2.04	0.658	6.07	6.13	0.889
*Surgery-related data*						
ASA grade [(n) %]			0.289			0.883
I	64 (15.88%)	250 (19.56%)		48 (16.11%)	51 (17.11%)	
II	174 (43.18%)	483 (37.79%)		119 (39.93%)	117 (39.26%)	
III	165 (40.94%)	545 (42.65%)		131 (43.96%)	130 (43.62%)	
mECM			0.570			0.773
< 0	12 (2.98%)	48 (3.76%)		9 (3.02%)	10 (3.36%)	
0	197 (48.88%)	663 (51.88%)		146 (48.99%)	147 (49.32%)	
1–5	58 (14.39%)	167 (13.07%)		42 (14.09%)	40 (13.42%)	
6–13	107 (26.55%)	350 (27.39%)		80 (26.85)	81 (27.18)	
≥ 14	29 (7.22%)	50 (3.91%)		21 (7.05%)	20 (6.71%)	
*In-hospital data*						
Preoperative Hb level (g/dL)	9.38 ± 2.12	9.97 ± 2.30	0.786	9.62 ± 2.23	9.68 ± 2.29	0.819
Preoperative VAS score	5.32 ± 1.68	6.29 ± 1.76	0.381	5.78 ± 1.69	5.97 ± 1.71	0.768
GDS	4.86 ± 1.37	4.03 ± 1.24	0.389	4.57 ± 1.33	4.51 ± 1.27	0.882
FIM	81.69 ± 9.85	86.98 ± 10.65	0.019	83.26 ± 9.89	84.39 ± 10.0	0.773

Values are presented as the number (%) or mean ± SD (standard deviation). BMI = body mass index; ASA = American Society of Anesthesiologists; mECM = modified Elixhauser’s Comorbidity Measure; VAS = Visual analog scores; NRS = Numerical rating scale; GDS = Geriatric Depression Scale; FIM = Functional independence measure

The results of prematching and postmatching, which include operation-related data, VAS and NRS at discharge, LOS, POD incidence, functional outcomes, and mortalities, are displayed in Table 2. Although the differences in the characteristics of VAS and NRS at discharge were significantly reduced between the two groups (*P* = 0.043 and 0.038 respectively), after matching, there were no statistically significant differences in the characteristics of each covariance (*P* = 0.849 and 0.739 respectively). It is worth noting that the disparities in POD rate and functional outcomes became significant after propensity score-based matching (*P* < 0.001 and 0.029, respectively); however, prior to matching, these differences were not statistically significant. No significant difference was observed in other operation-related data, LOS, and crude mortality rates at 30-day, 90-day, and 12-month before and after PSM.

**Table 2:** Patient outcome analyses before and after propensity score matching

Variables	Pre-matching			Post-matching		
	IBT group (*n* = 403)	Non-IBT group (*n* = 1278)	*P* value	IBT group (*n* = 298)	Non-IBT group (*n* = 298)	*P* value
Duration of operation (Mins)	96.58 ± 15.16	100.1 ± 19.37	0.493	98.46 ± 35.92	98.86 ± 36.01	0.887
VAS at discharge	2.98 ± 0.89	2.37 ± 0.61	0.043	2.67 ± 1.65	2.70 ± 1.68	0.849
NRS at discharge	15.39 ± 2.88	16.03 ± 3.02	0.038	14.6 ± 6.98	14.8 ± 7.02	0.739
LOS (day)	15.1 ± 2.74	14.6 ± 2.28	0.376	14.9 ± 5.92	14.8 ± 5.98	0.876
POD (Yes)	41 (9.93%)	106 (8.29%)	0.864	40 (13.42%)	23 (7.72%)	< 0.001
30-day mortality	4 (0.99%)	16 (1.25%)	0.658	4 (1.34%)	3 (1.01%)	0.927
90-day mortality	6 (1.49%)	22 (1.72%)	0.744	5 (1.68%)	4 (1.34%)	0.869
12-month mortality	28 (6.95%)	92 (7.20%)	0.813	21 (7.05%)	20 (6.71%)	0.902
Functional outcomes [(n) %]			0.749			0.029
Independent walking	161 (39.95%)	512 (40.06%)		119 (39.93%)	132 (44.30%)	
Use of walking aids	120 (29.78%)	384 (30.05%)		89 (29.87%)	104 (34.90%)	
Use of wheelchair	21 (5.21%)	64 (5.01%)		15 (5.03%)	10 (3.36%)	
Bedridden	21 (5.21%)	71 (5.56%)		16 (5.37%)	7 (2.35%)	
Death	80 (19.85%)	247 (19.33%)		59 (19.80%)	45 (15.10%)	

Values are presented as the number (%) or mean ± SD (standard deviation) or median (interquartile range). VAS = visual analog scores; NRS = numerical rating scores; LOS = Length of hospital stay; POD = postoperative delirium

To examine if IBT were correlated with POD and postoperative functional outcomes, correlation analyses were performed. By using the Spearman method, our results showed incidence of POD (*P* = 0.006) and functional outcomes (*P* = 0.013) were significantly associated with IBT (Tables 3**)**.

**Table 3:** The association of IBT with POD and postoperative functional outcomes

Variables	IBT group (*n* = 298)	Non-IBT group (*n* = 298)	Spearman’s *r* statistic	*P* value
LOS (day)	14.9 ± 5.92	14.8 ± 5.98		
POD (Yes)	40 (13.42%)	23 (7.72%)	− 0.29	0.006
30-day mortality	3 (1.01%)	4 (1.34%)	0.324	0.237
90-day mortality	4 (1.34%)	5 (1.68%)	0.297	0.335
12-month mortality	20 (6.71%)	21 (7.05%)	0.419	0.417
Functional outcomes [(*n*) %]			− 0.28	0.013
Independent walking	119 (39.93%)	132 (44.30%)		
Use of walking aids	89 (29.87%)	104 (34.90%)		
Use of wheelchair	15 (5.03%)	10 (3.36%)		
Bedridden	16 (5.37%)	7 (2.35%)		
Death	59 (19.80%)	45 (15.10%)		

Values are presented as the number (%) or mean ± SD (standard deviation) or median (interquartile range). LOS = Length of hospital stay; POD = postoperative delirium

## Discussion

POD is a prevalent complication and a significant contributor to post-surgical mortality, particularly in the context of hip surgery. Several studies have reported varying rates of POD among the elderly population in Asian countries, ranging from 2 to 7%, with even higher rates exceeding 50% following hip fracture repair [21, 22]. In a comprehensive multicentre observational study conducted by Unal D, it was found that 25.8% of patients received a peri-operative transfusion [16]. Despite efforts made by scholars to investigate the association between IBT and POD, the presence of numerous confounding variables within the data poses challenges to the validity and reliability of the results. In this study, data was gathered from a sample of 1681 patients, and PSM analysis was employed to investigate the association between IBT and POD. These findings indicate a significant correlation between IBT and an elevated occurrence of POD, it further hinders the postoperative functional recuperation of elderly patients with hip fracture.

Hip fractures in the elderly are a prevalent form of fracture, typically resulting from factors such as osteoporosis or unintentional falls among this demographic. The surgical intervention for hip fractures in the elderly entails a substantial procedure involving surgical trauma, anesthesia, intraoperative blood loss, pain, and other stimuli, all of which have the potential to induce postoperative delirium in patients [11, 23]. Furthermore, elderly patients frequently experience cognitive and memory decline, rendering them more susceptible to anxiety, fear, and other emotional responses when confronted with surgery and postoperative pain, consequently elevating the risk of adverse outcomes [24]. Geriatric individuals frequently present with prevalent underlying medical conditions, including hypertension, diabetes, and coronary heart disease, among others, which can potentially contribute to the development of postoperative delirium [25, 26].

Due to the pathological and physiological characteristics of elderly patients, blood transfusion is often required during surgery. However, the efficacy of intraoperative blood transfusion is not universally advantageous. The occurrence of postoperative cognitive impairment may be attributed to diminished cerebral blood flow, metabolic dysfunction, and an inflammatory reaction during surgical procedures. Remy KE et al. have posited that the administration of red blood cells can elicit a dual impact, both inhibiting and promoting inflammatory responses within the organism [27]. Similarly, Hensler et al. have suggested that the transfusion of blood products in patients with multiple injuries can activate cytokines and inflammatory mediators, such as tumor necrosis factor α and Interleukin-6, within the body [28]. Despite the limited number of clinical trials investigating the correlation between blood transfusion and postoperative delirium in elderly patients with hip fractures, it is noteworthy that no previous study has exclusively examined this association in the elderly population. Nevertheless, an Umbrella review encompassing 35 pertinent literature pieces indicates that intraoperative blood transfusion could potentially serve as an autonomous risk factor for postoperative delirium [29]. To consolidate the data, our study employed propensity score matching analysis. This study demonstrates a significant association between IBT and the occurrence of POD. The findings indicate that the incidence of POD is significantly increased with the administration of IBT. Moreover, the correlation analysis supports the consistent conclusion drawn from previous research.

With the progression of society and the elongation of human lifespan, population aging has emerged as a worldwide phenomenon. Due to the prevalence of osteoporosis among the elderly, the decline in bodily functions, and inadequate protection of hip joint muscles, even minor injuries can lead to hip fractures, resulting in a high incidence rate among the elderly. Consequently, the factors influencing postoperative functional recovery of the hip have consistently garnered attention from orthopedic physicians. Research has revealed that age, BMI, ASA grading, and anesthesia methods all serve as independent risk factors that impact the postoperative recovery of patients [30]. The frequency of intraoperative blood transfusion is intricately linked to the extent of preoperative anemia, intraoperative hemorrhage, and the patient's vital signs. While orthopedic and anesthesiologists continue to debate the optimal approach for blood transfusion during surgery for elderly patients with hip fractures, Leuzinger et al. have suggested that intraoperative blood transfusion may elevate the occurrence of postoperative pulmonary complications and prolong hospitalization in this patient population, albeit without impacting postoperative mortality rates [31].

Numerous scholarly works have examined the utilization of intra-operative blood transfusion as a means to address perioperative anemia in patients with hip fractures [31]. These studies have demonstrated a positive correlation between such transfusions and a reduction in POD rates [29], while also highlighting their potential negative impact on functional outcomes [32, 33]. Nevertheless, none of these investigations have accounted for potential confounding variables when reporting their findings. Thus, PSM analysis was applied in the current study to mitigate the impact of bias and confounding variables in the data, facilitating more valid comparisons between the experimental and control groups. Additionally, PSM analysis aids in data balancing and mitigates statistical errors arising from data imbalance. Our findings, derived from PSM analysis, reveal significant disparities in delirium rates and functional outcomes, even in the absence of notable distinctions between the initial two groups in terms of machinery. Recent studies have revealed that blood transfusion can potentially give rise to avoidable hazards or unfavorable responses, including circulatory overload, diminished cardiac output, acute renal impairment, hemolytic transfusion reactions, allergic responses, antibody production, infection, and mortality [34, 35]. Several research reports indicate that, in older patients who have undergone hip fracture surgery or have a higher cardiovascular risk, free transfusion strategies (transfusions with higher hemoglobin levels) do not result in reduced mortality or improved physical function recovery after discharge, when compared to restrictive transfusion strategies (transfusions based on hemoglobin levels). Our research findings also confirm that intraoperative blood transfusion significantly reduces the degree of functional recovery in elderly patients with hip fractures after surgery, and increases which consists with the current study [36].

It is evident that this study possesses certain limitations. Firstly, Due to its excellent analgesic effect and high patient acceptance rate, spinal anesthesia is a commonly used anesthesia method for elderly patients with intertrochanteric fractures. In order to eliminate the interference of anesthesia methods on experimental results, we only included elderly patients who received spinal anesthesia in this study. Secondly, the analysis was solely retrospective, encompassing a sample size of 1681 elderly patients with hip fractures within our unit over a four-year period. Consequently, findings derived from studies conducted over longer durations and across multiple centers may yield more compelling results. Thirdly, we did not undertake a stratified analysis to examine the correlation between the quantity of intraoperative blood transfusion and the occurrence of postoperative delirium in a systematic manner. Finally, we did not explore the potential impacts of preoperative blood transfusion and intraoperative infusion of other blood products, such as platelets, plasma, and cryoprecipitates.

## Conclusion

In conclusion, IBT seems to increase the risk of POD and hinder functional recovery in elderly patients with hip fracture. Further studies should focus on choosing the appropriate timing for blood transfusion for such patients to achieve better postoperative outcomes.

## Data Availability

The datasets used and/or analyzed during the current study are available from the corresponding author on reasonable request.

## Abbreviations

POD: Postoperative delirium
ASA: American Society of Anesthesiologists
IBT: Intra-operative blood transfusion
LOS: Length of hospital stay
PFNA: Proximal femoral nail antirotation
MMSE: Mini-mental state examination
BIS: Bispectral index
ETCO_2_: End-tidal carbon dioxide
BMI: Body mass index
mECM: Modified Elixhauser comorbidity measures
VAS: Visual analog scores
NRS: Numerical rating scores
CAM-CR: The Confusion Assessment Method-Chinese Revision
GDS: Geriatric Depression Scale
FIM: Functional independence measure
SD: Standard deviation
IQR: Interquartile range
PSM: Propensity score matching

## References

[1] Oh ES, Fong TG, Hshieh TT, Inouye SK (2017) Delirium in older persons: advances in diagnosis and treatment. JAMA 318:1161–1174 28973626 10.1001/jama.2017.12067 PMC5717753

[2] Ma X, Chu H, Han K, Shao Q, Yu Y, Jia S, Wang D, Wang Z, Zhou Y (2023) Postoperative delirium after transcatheter aortic valve replacement: An updated systematic review and meta‐analysis. J Am Geriatr Soc 71:646–660 36419366 10.1111/jgs.18104

[3] Ho MH, Nealon J, Igwe E, Traynor V, Chang HR, Chen KH, Montayre J (2021) Worldviews Evid-based Nurs 18:290–301 34482593 10.1111/wvn.12536

[4] Migirov A, Chahar P, Maheshwari K (2021) Postoperative delirium and neurocognitive disorders. Curr Opin Crit Care 27:686–693 34545028 10.1097/MCC.0000000000000882

[5] Oh ST, Park JY (2019) Korean J Anesthesiol 72:4–12 30139213 10.4097/kja.d.18.00073.1 PMC6369344

[6] Swarbrick CJ, Partridge JSL (2022) Anaesthesia 77(Suppl 1):92–101 35001376 10.1111/anae.15607

[7] Jin Z, Hu J, Ma D (2020) Br J Anaesth 125:492–504 32798069 10.1016/j.bja.2020.06.063

[8] Wu J, Yin Y, Jin M, Li B (2021) Int J Geriatr Psychiatry 36:3–14 32833302 10.1002/gps.5408

[9] Veronese N, Maggi S (2018) Injury 49:1458–1460 29699731 10.1016/j.injury.2018.04.015

[10] Parker M, Johansen A (2006) BMJ (Clinical research ed) 333:27–30 16809710 10.1136/bmj.333.7557.27 PMC1488757

[11] Chen Y, Liang S, Wu H, Deng S, Wang F, Lunzhu C, Li J (2022) Front Aging Neurosci 14:1068278 36620772 10.3389/fnagi.2022.1068278 PMC9813601

[12] Ziranu A, Meschini C, De Marco D, Sircana G, Oliva MS, Rovere G, Corbingi A, Vitiello R, Maccauro G, Pola E (2022) Orthopedic reviews 14:38574 36267213 10.52965/001c.38574 PMC9568419

[13] S.R. Lewis, M.W. Pritchard, L.J. Estcourt, S.J. Stanworth, X.L. Griffin, The Cochrane database of systematic reviews, 6 (2023) Cd013737. 10.1002/14651858.CD013737.pub2 PMC10249061 37294864

[14] M.S. Dawod, M.S. Alisi, Y.O. Saber, Q.A. Abdel-Hay, B.M. Al-Aktam, Y. Alfaouri, L.B. Alfraihat, A.A. Albadaineh, A.Z. Abuqudiri, R.M. Odeh, A.A.R. Altamimi, M.A. Alrawashdeh, M.M. Alebbini, O.A. Abu-Dhaim, A.A. Al-Omari, I. Alaqrabawi, M.N. Alswerki, A. Abuawad, M.R. Al Nawaiseh, Y. Hammad, J. Al-Ajlouni, International journal of general medicine, 15 (2022) 6591–6598. 10.2147/IJGM.S373313 PMC9385130 35991940

[15] W. Wan, L. Li, Z. Zou, W. Chen, European geriatric medicine, (2024). 10.1007/s41999-024-01095-7 39499481

[16] D. Unal, Y. Senayli, R. Polat, D.R. Spahn, F. Toraman, N. Alkis, A. Zekeriyya, A. Bahar, B.A. Onat, B. Hulya, B. Mehmet, C. Nesil, D. Asli, G. Suna, G.C. Meltem, O. Mukadder, S. Mert, T. Busra, T.H. Ilksen, Y.G. Cigdem, A. Suheyla, A. Yesim, A.E. Arzu, A.T. Esra, A. Ali, A. Mine, A. Nukhet, A.S. Mustafa, A. Ergin, A.M. Cavidan, A. Sule, A.A. Gulbin, A. Emine, A. Esma, A. Mahmut, A. Necmiye, A. Zuhal, A. Hilal, B. Cumhur, B. Volkan, B. Nurdan, B. Azize, B. Zekiye, B.M. Ugur, B.O. Faruk, B. Sibel, C.T. Sanem, C. Meltem, C. Baris, C. Ayse, C. Zubeyir, C.E.F. Banu, C. Faruk, C. Alkin, C.Y. Ziya, D. Esra, D.H. Fisun, D. Abdurrahim, D.O. Ayca, E. Osman, E.K. Gulay, E. Engin, E. Ipek, A.E. Tekeli, G. Mehmet, G. Basak, G. Gamze, G. Emel, G. Isin, G.A. Betul, H. Gulcin, H.S. Nazan, I.K. Ozgen, I.E. Ayse, I. Muzeyyen, K. Inci, K. Deniz, K. Derya, K. Arzu, K.A. Duygu, K. Mensure, K. Suleyman, K.B. Zuleyha, K. Gulsen, K. Oya, K. Yeliz, K. Pakize, K. Zeynep, K. Ceren, K. Betul, K. Semih, K. Gamze, K. Ilke, K. Aysun, K. Omer, M.B. Ceyda, N. Burak, O.R. Dilek, O. Dilek, O. Yavuz, O. Elif, Ö. Esra, O. Menekse, O.E. Sabri, O. Yetkin, O. Aysegul, O.M. Ozgur, O. Onur, S. Ozlem, S. Arzu, S. Aslinur, S. Mehmet, S. Cihan, S. Yeliz, S. Nevriye, S. Ayten, S.K. Tolga, S. Hilal, S.S. Gokce, S. Betul, S. Ozlem, S.E. Bengi, S.F. Isil, S. Emin, S.F. Dilek, T.K. Ebru, T. Nilay, T.Z. Tuncel, T. Sibel, T.K. Gonul, T. Hulya, T. Abdurrahman, U. Fatih, U. Canan, U. Petek, U. Suheyla, U.S. Gulcin, U. Filiz, Y.A. Selcan, Y. Kadir, Y. Mustafa, Y.A. Aysun, Y. Munise, Y.E. Hatice, Y. Hakan, Y. Mehmet, Y. Nureddin, Blood transfusion = Trasfusione del sangue, 18 (2020) 261–279. 10.2450/2020.0011-20 PMC7375885 32697928

[17] Mears SC (2014) Clin Geriatr Med 30:229–241 24721363 10.1016/j.cger.2014.01.004

[18] Ji HM, Han J, Bae HW, Won YY (2017) BMC Musculoskelet Disord 18:375 28854917 10.1186/s12891-017-1738-3 PMC5577758

[19] Schreiner AS, Hayakawa H, Morimoto T, Kakuma T (2003) Int J Geriatr Psychiatry 18:498–505 12789670 10.1002/gps.880

[20] Goto K, Kataoka H, Honda A, Yamashita J, Morita K, Hirase T, Sakamoto J, Okita M (2020) Pain Res Manage 2020:8814290 10.1155/2020/8814290 PMC7657670 33204378

[21] Choi YH, Kim DH, Kim TY, Lim TW, Kim SW, Yoo JH (2017) Clin Interv Aging 12:1835–1842 29138544 10.2147/CIA.S147585 PMC5680947

[22] Sahadevan S, Saw SM, Gao W, Tan LC, Chin JJ, Hong CY, Venketasubramanian N (2008) J Am Geriatr Soc 56:2061–2068 19016940 10.1111/j.1532-5415.2008.01992.x

[23] Bai J, Liang Y, Zhang P, Liang X, He J, Wang J, Wang Y (2020) Osteoporosis international : a journal established as result of cooperation between the European Foundation for Osteoporosis and the National Osteoporosis Foundation of the USA 31:317–326 31741024 10.1007/s00198-019-05172-7

[24] Yang KL, Detroyer E, Van Grootven B, Tuand K, Zhao DN, Rex S, Milisen K (2023) BMC Geriatr 23:198 36997928 10.1186/s12877-023-03923-0 PMC10064748

[25] Gracie TJ, Caufield-Noll C, Wang NY, Sieber FE (2021) Anesth Analg 133:314–323 34257192 10.1213/ANE.0000000000005609 PMC8289124

[26] Hung KC, Wang LK, Lin YT, Yu CH, Chang CY, Sun CK, Chen JY (2022) J Clin Anesth 79:110681 35255352 10.1016/j.jclinane.2022.110681

[27] Remy KE, Hall MW, Cholette J, Juffermans NP, Nicol K, Doctor A, Blumberg N, Spinella PC, Norris PJ, Dahmer MK, Muszynski JA (2018) Transfusion 58:804–815 29383722 10.1111/trf.14488 PMC6592041

[28] T. Hensler, B. Heinemann, S. Sauerland, R. Lefering, B. Bouillon, J. Andermahr, E.A. Neugebauer, Shock (Augusta, Ga.), 20 (2003) 497–502. 10.1097/01.shk.0000095058.62263.1f 14625472

[29] P. Bramley, K. McArthur, A. Blayney, I. McCullagh, International journal of surgery (London, England), 93 (2021) 106063. 10.1016/j.ijsu.2021.106063 34411752

[30] W. Chang, H. Lv, C. Feng, P. Yuwen, N. Wei, W. Chen, Y. Zhang, International journal of surgery (London, England), 52 (2018) 320–328. 10.1016/j.ijsu.2018.02.061 29530826

[31] Leuzinger E, Poblete B, Konrad CJ, Hansen D (2018) Eur J Anaesthesiol 35:972–979 30234668 10.1097/EJA.0000000000000883

[32] Crowther M, van der Spuy K, Roodt F, Nejthardt MB, Davids JG, Roos J, Cloete E, Pretorius T, Davies GL, van der Walt JG, van der Westhuizen C, Flint M, Swanevelder JLC, Biccard BM (2018) Anaesthesia 73:812–818 29529331 10.1111/anae.14239

[33] S.J. Brunskill, S.L. Millette, A. Shokoohi, E.C. Pulford, C. Doree, M.F. Murphy, S. Stanworth, The Cochrane database of systematic reviews, (2015) Cd009699. 10.1002/14651858.CD009699.pub2 PMC11065123 25897628

[34] Myers A, Frank I, Shah PH, Tarrell RF, Baird B, Dora C, Karnes RJ, Thompson RH, Tollefson MK, Boorjian SA, Lyon TD (2023) J Urol 209:525–531 36445045 10.1097/JU.0000000000003094

[35] Lu Q, Zhang J, Gao WM, Lv Y, Zhang XF, Liu XM (2018) Medical science monitor : international medical journal of experimental and clinical research 24:8469–8480 30470732 10.12659/MSM.910978 PMC6270889

[36] Carson JL, Terrin ML, Noveck H, Sanders DW, Chaitman BR, Rhoads GG, Nemo G, Dragert K, Beaupre L, Hildebrand K, Macaulay W, Lewis C, Cook DR, Dobbin G, Zakriya KJ, Apple FS, Horney RA, Magaziner J (2011) N Engl J Med 365:2453–2462 22168590 10.1056/NEJMoa1012452 PMC3268062

